# Efficacy of different plant extracts in the prevention of radiation dermatitis in radiotherapy patients with nasopharyngeal carcinoma: A protocol for a Bayesian network meta-analysis

**DOI:** 10.1097/MD.0000000000023523

**Published:** 2020-12-18

**Authors:** Yi Yi, Xingli You, Ying Long, Ya Huang

**Affiliations:** a Department of Otolaryngology, The Second Branch of Chongqing Ninth People's Hospital; b Department of Medical Insurance, Chongqing Fifth People's Hospital; c Department of Oncology, Chongqing Ninth People's Hospital, Chongqing, China.

**Keywords:** meta-analysis, nasopharyngeal carcinoma: a Bayesian, plant extracts, radiation dermatitis, radiotherapy

## Abstract

**Background:**

Radiation dermatitis is a common complication in patients with nasopharyngeal carcinoma (NPC) when treated with radiotherapy. Plant extracts have good effects on the prevention of radiation dermatitis in patients with NPC when treated with radiotherapy. However, there is insufficient comparison among the currently used plant extracts. Therefore, the purpose of this study was to explore the efficacy of different plant extracts in the prevention of radiation dermatitis in patients with NPC by Bayesian network meta-analysis.

**Methods:**

We searched Chinese and English databases to collect all randomized controlled trials (RCT) of plant extracts for the prevention of radiation dermatitis in NPC patients who underwent radiotherapy up to October 2020. Two researchers then independently screened articles, extracted data and evaluated the quality selected literatures. All data were processed by Stata 14.0 and WinBUGS V.1.4.3.

**Results:**

We applied Bayesian statistical model for network meta-analysis, ranked the effects of various plant extracts, and adopted GRADE method to explain the results.

**Conclusion:**

Our study is expected to provide high-quality evidence-based medicine advice for the prevention of radiation dermatitis in patients suffering from NPC undergoing radiotherapy.

**Ethics and dissemination:**

Ethical approval was not required for this study. The systematic review will be published in a peer-reviewed journal, presented at conferences, and will be shared on social media platforms. This review would be disseminated in a peer-reviewed journal or conference presentations.

**OSF REGISTRATION NUMBER:**

DOI 10.17605/OSF.IO/6SV45.

## 1 Introduction

At present, cancer mortality has become the leading cause of death, and nearly 50% of cancer patients need radiotherapy.^[1]^ Nasopharyngeal carcinoma (NPC) is the most common malignant tumor in head and neck squamous cell carcinoma, and frequently occurs in southern China and Southeast Asia, due to Epstein-Barr virus (EBV) infection. NPC is highly sensitive to radiotherapy, so it is the first option for the treatment of NPC. Radiation dermatitis is one of the common side effects during radiotherapy, and up to 95% of patients may experience this side effect.^[2]^

Although radiotherapy techniques continue to improve, such as the application of three-dimensional shape-adapted intensity modulated radiotherapy, skin reaction still remains inevitable. It was reported that 85% of radiotherapy patients develop skin reactions from local erythema to wet desquamation that not only increases the pain and hospitalization costs of patients, but also leads to the interruption of radiotherapy.^[3]^ More prophylactic drugs can be used for clinical epithelial reaction, but its management is often based on personal opinions or experience, rather than evidence-based practice.^[4]^ Effective clinical practice guidelines are insufficient on the application of preventive drugs.^[5]^ Therefore, to find a safe, efficient, cheap, and affordable way to prevent or treat radiation dermatitis has become a strong demand of the majority of clinical medical workers and patients.

Plant extract refers to one kind of substance extracted or processed from all or a part of plant through appropriate solvents or methods.^[6]^ However, how to choose the appropriate plant extract has become an urgent problem and need to be solved clinically. Traditional meta-analysis can only achieve pairwise comparison among drugs, while Network meta-analysis can be adopted to quantitatively analyze >10 kinds of intervention measures for same diseases. And ranking the probability of advantages and disadvantages based on different outcome indicators can help clinicians choose the best scheme among different intervention measures. Therefore, according to the process of systematic review and meta-analysis priority report items, this study was evaluated by Bayesian network meta-analysis, so as to provide a basis for the clinical evaluation of plant extracts in the prevention and treatment of radiation dermatitis of NPC.

## 2 Methods

### 2.1 Study registration

The protocol of this review was registered in OSF (OSF registration number: DOI 10.17605/OSF.IO/6SV45). It was reported to follow the statement guidelines of preferred reporting items for systematic reviews and meta-analyses protocol.^[7]^

### 2.2 Inclusion criteria for study selection

#### 2.2.1 Types of studies

A randomized controlled trial (RCT) was enrolled to investigate the effects of plant extracts in the prevention of radiation dermatitis in radiotherapy patients with NPC published in Chinese and English.

Non-RCTs, cohort studies, case reports, experimental studies, and the data of the included study are missed or incomplete, and duplicate publications were excluded.

#### 2.2.2 Types of participants

(1) NPC was diagnosed through pathology or histology, and the age and race of the patients were not limited.
(2) Patients accepting radiotherapy.

#### 2.2.3 Types of interventions

Plant extracts or placebos.

#### 2.2.4 Types of outcome measures

The incidence of mild, moderate, and severe radiation dermatitis and the total incidence of radiation dermatitis were chosen as the evaluation criteria of the outcome.

### 2.3 Data sources

PubMed, Web of Science, Cochrane Library, EMBASE, Wan fang Database, Chinese Scientific Journal Database, China National Knowledge Infrastructure Database and Chinese Biomedical Literature Database were systematically searched. The time for literature retrieval is set to build the database until October 2020.

### 2.4 Searching strategy

The details of PubMed's search strategies are illustrated in Table 1, including all search terms, while similar search strategies are applied to other electronic databases.

**Table 1:** Search strategy (PubMed).

Number	Search terms
1	Plant Extracts[MeSH]
2	Extracts, Plant[Title/Abstract]
3	OR/1–2
4	Radiation dermatitis[Title/Abstract]
5	Nasopharyngeal Neoplasms[MeSH]
6	Cancer of Nasopharynx[Title/Abstract]
7	Nasopharyngeal Cancer[Title/Abstract]
8	Cancer of the Nasopharynx[Title/Abstract]
9	Nasopharynx Cancer[Title/Abstract]
10	Nasopharynx Neoplasms[Title/Abstract]
11	Neoplasms, Nasopharyngeal[Title/Abstract]
12	Cancer, Nasopharyngeal[Title/Abstract]
13	Cancer, Nasopharynx[Title/Abstract]
14	Cancers, Nasopharyngeal[Title/Abstract]
15	Cancers, Nasopharynx[Title/Abstract]
16	Nasopharyngeal Cancers[Title/Abstract]
17	Nasopharyngeal Neoplasm[Title/Abstract]
18	Nasopharynx Cancers[Title/Abstract]
19	Nasopharynx Neoplasm[Title/Abstract]
20	Neoplasm, Nasopharyngeal[Title/Abstract]
21	Neoplasm, Nasopharynx[Title/Abstract]
22	Neoplasms, Nasopharynx[Title/Abstract]
23	OR/5–22
24	3 AND 4 AND 23

### 2.5 Data collection and analysis

#### 2.5.1 Literature screening and data extraction

**Figure 1:**
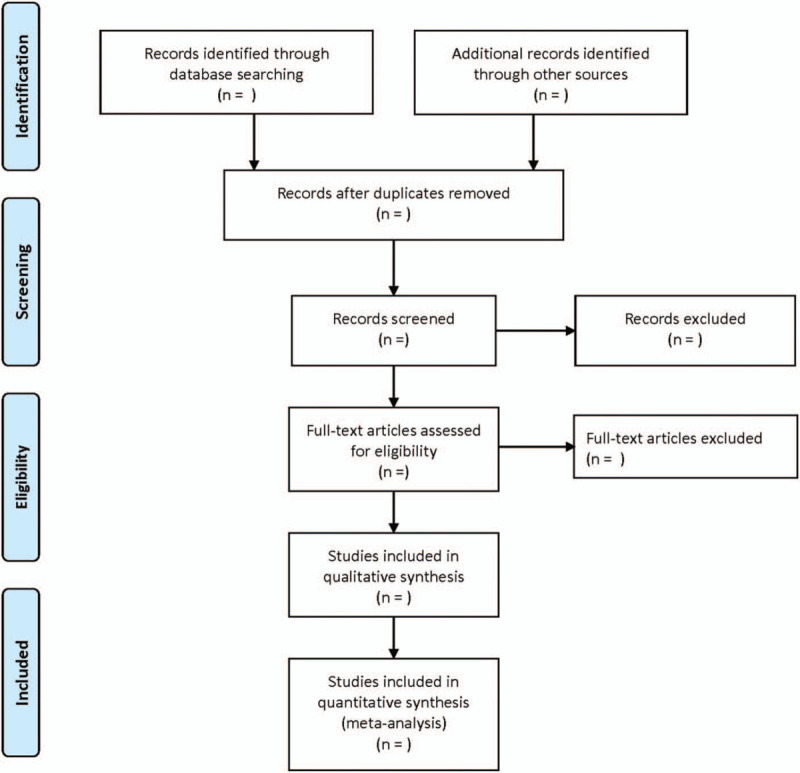
Flow diagram showing literature filtration process.

According to the inclusion and exclusion criteria, 2 researchers independently completed the literature screening. By read through the full text, the data were extracted, and the final results were cross-checked. If there are different opinions, it would be further negotiated and arbitrated with the third researcher. The extraction contents include: basic information included in the study, such as the year of publication, the place of literature, country, author, etc. Research methods include random method, sample size, research object, and blind method selection. Intervention measures of the 2 groups of patients. Related outcome indicators. The screening flow chart of this study is demonstrated in Fig. 1.

#### 2.5.2 Assessment of risk of bias

According to the bias risk assessment tool recommended by Cochrane hand book, 2 researchers evaluated literatures. The contents of the evaluation include: whether the generation of the random sequence is rigorous, whether the distribution scheme is hidden, what is the implementation of the blind method, whether the results are selectively reported, whether the outcome data are complete and other possible sources of bias (such as testing the outcome evaluation), and whether the 2 groups of baselines are balanced. The degree of bias wind risk is expressed with “yes,” “no,” and “unclear.” The evaluation grade is divided into A level, B level, and C level. Each of the included studies is rated as Grade A. Some of the above criteria are rated as Grade B. If the above criteria are not met, they are rated as Grade C.

#### 2.5.3 Measures of treatment effect

The dichotomous outcomes were estimated by the risk ratio (RR), with 95% confidence intervals (CIs).

#### 2.5.4 Management of missing data

If any data is missing, requesting the original data by email. If the missing data cannot be obtained, the data could be excluded from the study.

#### 2.5.5 Assessment of heterogeneity and data synthesis

Related charts were drawn with Stata 14.0 software (Stata Corp, College Station, TX). Based on Bayesian framework, WinBUGS 1.4.3 software (MRC Biostatistics Unit, Cambridge, UK) is applied to analyze the data. Bayesian inference is carried out by adopting Markov Chain-Monte Carlo (MCMC). According to the prior probability, a posteriori probability is inferred, and the estimation and inference were conducted on the assumption that MCMC has reached a stable convergence state. When running WinBUGS program, the number of iterations is set to 100,000, the first 10,000 are used for annealing, so as to eliminate the influence of the initial value, and the simulation chain is 3. According to the cumulative ranking probability map, the area size displays the probability ranking of each intervention as the best intervention. Heterogeneity test: *Q* test was applied to qualitatively determine inter-study heterogeneity: If *P* ≥ .1, there is no inter-study heterogeneity, while if *P* < .1, there is inter-study heterogeneity. Meanwhile, *I*^2^ value was adopted to quantitatively evaluate the inter-study heterogeneity: if *I*^2^ ≤ 50%, the heterogeneity is considered to be good, and the fixed-effect model would be adopted. If *I*^2^ > 50%, it indicates significant heterogeneity, and the source of heterogeneity would be explored through subgroup analysis or sensitivity analysis. If there is no obvious clinical or methodological heterogeneity, it would be considered as statistical heterogeneity, and the random-effect model would be applied for analysis. If there is significant clinical heterogeneity between the 2 groups, descriptive analysis would be used, while subgroup analysis is not required.

#### 2.5.6 Assessment of reporting biases

“Comparison-adjusted” funnel plot was drawn to evaluate publication bias.

#### 2.5.7 Subgroup analysis

When heterogeneity is discovered (such as intervention time and types of plant extracts), subgroup analysis would be applied to find out the source of heterogeneity.

#### 2.5.8 Sensitivity analysis

Through the study of large weight of elimination effect, the sensitivity analysis was performed to test the stability of the results of meta-analysis.

#### 2.5.9 Grading the quality of evidence

We used GRADE to evaluate the quality of evidence from the following 5 aspects: risk of bias, indirectness, inconsistency, imprecision, and publication bias.^[8]^

#### 2.5.10 Ethics and dissemination

The content of this article does not involve moral approval or ethical review and would be presented in print or at relevant conferences.

## 3 Discussion

Radiation dermatitis is an inflammatory reaction of skin and mucosa and caused by β-ray, γ-ray, and x-ray.^[9,10]^ Skin is one of the tissues with moderate sensitivity to radiation. The mechanism of skin radiation injury is the reflex dilatation of capillaries in the radiation field, thus resulting in local congestive reaction, erythema, vascular injury, microcirculation disturbance, and even the formation of skin ulcers.^[11]^ Progressive microvascular obstruction and dysplasia of epithelial cells and fibroblasts are all important causes of poor wound healing.

Despite the remarkable development of radiotherapy technology, effective intervention measures are still insufficient in the prevention of radiation dermatitis. The current evidence does not provide sufficient sound guidelines. Many drugs and dressings can only treat the side effects of radiotherapy, but cannot prevent them. In view of the high incidence of dermatitis during radiotherapy and the serious negative impact on the quality of life, it is very important to prevent and manage the side effects of radiation dermatitis.^[12]^ Although there are some studies advocating protective measures such as topical application, dressing, or phototherapy to improve side effects, the lack of evidence supports the application of protective measures, including topical agents, dressings, or phototherapy.^[13]^

At present, up to 50% of dermatosis patients take complementary and alternative drugs, including botanical preparations, usually combined with traditional drugs.^[14–17]^ Based on the available evidence, our study can provide the best plant extract for the prevention of radiation dermatitis in patients with NPC after radiotherapy. However, the ranking results should be treated cautiously, should be verified by RCT with reasonable design and rigorous methodology, thus providing recommendations for clinical selection.

## Author contributions

**Data curation:** Yi Yi.

**Formal analysis:** Yi Yi.

**Funding acquisition:** Yi Yi.

**Methodology:** Xingli You.

**Project administration:** Yi Yi.

**Software:** Ying Long.

**Supervision:** Yi Yi.

**Validation:** Ya Huang.

**Visualization:** Ying Long.

**Writing – original draft:** Yi Yi, Xingli You.

**Writing – review & editing:** Yi Yi.

## References

[1] Chen W Zheng R Zhang S Cancer incidence and mortality in China in 2013: an analysis based on urbanization level. Chin J Cancer Res 2017;29:1–0. 28373748 10.21147/j.issn.1000-9604.2017.01.01 PMC5348470

[2] Ryan JL Ionizing radiation: the good, the bad, and the ugly. J Invest Dermatol 2012;132:985–93. 22217743 10.1038/jid.2011.411 PMC3779131

[3] Glover D Harmer V Radiotherapy-induced skin reactions: assessment and management. Br J Nursing 2014;23:S28 S30–S35. 10.12968/bjon.2014.23.Sup2.S28 24619051

[4] Nystedt KE Hill JE Mitchell AM The standardization of radiation skin care in British Columbia: a collaborative approach. Oncol Nurs Forum 2005;32:1199–205. 16270115 10.1188/05.ONF.1199-1205

[5] Zhang Y Zhang S Shao X Topical agent therapy for prevention and treatment of radiodermatitis: a meta-analysis. Support Care Cancer 2013;21:1025–31. 23064885 10.1007/s00520-012-1622-5

[6] Rafati M Ghasemi A Saeedi M Nigella sativa L. for prevention of acute radiation dermatitis in breast cancer: a randomized, double-blind, placebo-controlled, clinical trial. Complement Ther Med 2019;47:102205. 31780017 10.1016/j.ctim.2019.102205

[7] Shamseer L Moher D Clarke M Preferred reporting items for systematic review and meta-analysis protocols (PRISMA-P) 2015: elaboration and explanation. BMJ 2015;350:g7647. 25555855 10.1136/bmj.g7647

[8] Guyatt GH Oxman AD Vist GE GRADE: an emerging consensus on rating quality of evidence and strength of recommendations. BMJ 2008;336:924–6. 18436948 10.1136/bmj.39489.470347.AD PMC2335261

[9] Rades D Narvaez CA Doemer C Radiotherapy-related skin toxicity (RAREST-02): A randomized trial testing the effect of a mobile application reminding head-and-neck cancer patients to perform skin care (reminder app) on radiation dermatitis. Trials 2020;21:424. 32450921 10.1186/s13063-020-04307-0 PMC7249413

[10] Ashack KA Kuritza V Visconti MJ Dermatologic sequelae associated with radiation therapy. Am J Clin Dermatol 2020;21:541–55. 32410134 10.1007/s40257-020-00519-x

[11] Schmeel LC Koch D Schmeel FC Acute radiation-induced skin toxicity in hypofractionated vs. conventional whole-breast irradiation: an objective, randomized multicenter assessment using spectrophotometry. Radiother Oncol 2020;146:172–9. 32171945 10.1016/j.radonc.2020.02.018

[12] Benomar S Boutayeb S Lalya I [Treatment and prevention of acute radiation dermatitis]. Cancer Radiother 2010;14:213–6. 20427219 10.1016/j.canrad.2010.02.001

[13] Schmidt FMQ González CVS Mattar RC [Clinical protocol for the topical use of a cream with nanoparticles and vitamin E to prevent radiodermatitis in patients with breast cancer]. J Wound Care 2020;29:18–26. 10.12968/jowc.2020.29.LatAm_sup_1.18 31859604

[14] Baron SE Goodwin RG Nicolau N Use of complementary medicine among outpatients with dermatologic conditions within Yorkshire and South Wales, United Kingdom. J Am Acad Dermatol 2005;52:589–94. 15793507 10.1016/j.jaad.2004.11.058

[15] Fuhrmann T Smith N Tausk F Use of complementary and alternative medicine among adults with skin disease: updated results from a national survey. J Am Acad Dermatol 2010;63:1000–5. 20933300 10.1016/j.jaad.2009.12.009

[16] Jensen P Use of alternative medicine by patients with atopic dermatitis and psoriasis. Acta Derm Venereol 1990;70:421–4. 1980977

[17] Smith N Shin DB Brauer JA Use of complementary and alternative medicine among adults with skin disease: results from a national survey. J Am Acad Dermatol 2009;60:419–25. 19157642 10.1016/j.jaad.2008.11.905

